# Carbon Ion Dose Constraints in the Head and Neck and Skull Base: Review of MedAustron Institutional Protocols

**DOI:** 10.14338/IJPT-20-00093.1

**Published:** 2021-06-25

**Authors:** Piero Fossati, Ana Perpar, Markus Stock, Petra Georg, Antonio Carlino, Joanna Gora, Giovanna Martino, Eugen B. Hug

**Affiliations:** 1 MedAustron Ion Therapy Center, Wiener Neustadt, Austria; 2 Oncology Institute Ljubljana, Ljubljana, Slovenia

**Keywords:** dose constraints, RBE models, carbon ion radiotherapy

## Abstract

**Background:**

Dose constraints are of paramount importance for the outcome of any radiotherapy treatment. In this article, we report dose-volume constraints as well as currently used fractionation schedules for carbon ion radiotherapy as applied in MedAustron (Wiener Neustadt, Austria).

**Materials and Methods:**

For fractionation schedules, both German and Japanese regimes were used. From the clinical experience of National Institute of Radiological Sciences (Chiba, Japan) and Heidelberg Ion Therapy (Heidelberg, Germany; formerly GSI Helmholtzzentrum für Schwerionenforschung, Darmstadt, Germany) and the work by colleagues in Centro Nazionale Adroterapia Oncologica (Pavia, Italy) recalculating the dose from the microdosimetric kinetic model to the local effect model, we have set the dose constraints for critical organs of the head and neck area. Where no clinical data was available, an educated guess was made, based on data available from photon and proton series.

**Results:**

We report the constraints for the optic nerve and chiasm, brainstem, spinal cord, cochlea, brain parenchyma, salivary gland, eye and adnexa, and mandibular/maxillary bone; constraints are grouped based on a fractionation scheme (German versus Japanese) and the risk of toxicity (safe, low to middle, and middle to high).

**Conclusion:**

We think validation of dose constraints should present a relevant part of the activity of any carbon ion radiotherapy facility, and we anticipate future multicentric, joint evaluations.

## Introduction

Dose constraints to the organs at risk (OARs) are of paramount importance for the outcome of any radiotherapy (RT) treatment. Too-lax constraints can increase the risk of unwanted side effects and ultimately compromise the patient's quality of life; on the other hand, too-strict constraints will result in suboptimal target coverage and, therefore, in a reduced probability of local control. Dose constraints are as important as dose prescription in the attempt to achieve the uncomplicated cure of cancer.

Dose constraints in radiation oncology have been mostly determined by empirical clinical practice of radiotherapy centers. A historical milestone was the publication of the article on tolerance doses of OARs by Emami et al. [1] in 1991. Since then, it has become apparent that constraints have to be validated by long-term clinical-toxicity data. This led, in 2010, to a large, cooperative effort: the Quantitative Analysis of Normal Tissue Effect in the Clinics (QUANTEC) [2]. In the past 10 years, knowledge about dose constraints has constantly progressed. With highly conformal RT techniques becoming widely available, the concept of tolerance dose has evolved from simple numeric values (typically the mean or maximum dose) to more-complex volumetric or dose-volume histogram constraints. For selected endpoints, long-term clinical validation is so reliable that it is possible to predict the risk of toxicity (normal tissue complication probability [NTCP]) of any given radiotherapy plan with an accuracy that is sufficient for clinical decision-making. The best known example is the NTCP model for dysphagia after radiotherapy for head and neck squamous cell carcinoma [3].

Proton therapy is biologically and qualitatively different from photon-based RT. The order of magnitude of that difference is, however, quite small. Both the International Commission on Radiation Units and Measurements (ICRU) recommendations and widespread clinical practice agree on describing proton's radiobiological properties with a constant scaling factor of 1.1 (relative radiobiological effectiveness [RBE]) [4].

As a first approximation, the same constraints are used in proton therapy and photon-based intensity-modulated RT (IMRT). As an example, a more-detailed recommendation on dose constraints for proton therapy in neuro-oncology from the European Particle Therapy Network confirmed the applicability of photon dose constraints [5].

The major difference (ie, a more-permissive dose constraint for brainstem and optic pathways) is related to spatial-dose distribution and clinical experience of proton centers treating skull-base tumors, rather than to radiobiologic issues. The possibility of extrapolating photon experience to protons is so widely accepted that colleagues from the Netherlands are using NTCP modeling to select patients for IMRT or proton therapy with the so called model-based approach [6].

A more challenging task is adapting dose constraints derived from the mainstream 3-dimensional (3D) conformal RT/IMRT experience with normofractionation to stereotactic hypofractionated treatment. It is generally agreed that relying on a radiobiologic model to convert normofractionated constraints to a stereotactic body RT (SBRT) schedule is suboptimal and that specific SBRT constraints should be derived from clinical practice [7].

Carbon ion RT (CIRT) has been used in clinical practice for more than 20 years but is still available in only a few select centers. Similar to SBRT, CIRT is delivered with hypofractionated schedules. According to clinical practice and to ICRU recommendation, an RBE-weighted dose is used for prescribing and reporting the dose [8].

The RBE models used in CIRT are more complex than the simple scaling factor used in proton therapy. The CIRT RBE varies voxel by voxel and can be as high as 4 to 5. According to ICRU recommendations, any validated RBE model can be used, as long as it is clearly specified which one has been selected.

A combination of complex RBE models, hypofractionation, and the comparatively few patients treated makes determining dose constraints in CIRT a complex issue.

In this article, we will review dose constraints used at MedAustron Ion Therapy Center (Wiener Neustadt, Austria) for CIRT in the treatment of head and neck tumors, and we will discuss the underlying approach.

## CIRT RBE Models

When CIRT is used in clinical practice, the patient is exposed to a mixed field of particles consisting of carbon ion and secondary fragments of different energies. Part of the dose is deposited by densely ionizing particles (mainly primary carbon ions slowing down near the end of their path) and part is from sparsely ionizing particles. The densely ionizing component (also described as high linear energy transfer or the high LET component) has greater efficacy in cell killing. To mitigate the inhomogeneity in biological effect, the RBE is introduced. The RBE refers to a well-defined, measurable endpoint (typically cell survival of cell lines in vitro) compared with the effect of a reference radiation (typical photons). The RBE is computed voxel by voxel. Planning is performed to achieve the homogeneity of absorbed dose-X RBE over the target volumes.

In the Japanese experience, the Kanai semiempirical model was initially used for passive-scattered CIRT [9]. Subsequently, the modified microdosimetric kinetic model (MKM) was introduced for both passive scattering and active scanning, and consistency with the old model was kept [10]. In this article, we will refer to the Japanese RBE model simply as MKM.

In the European and Chinese experience, the RBE model used was the local effect model (LEM) [11].

Several versions of LEM are available (from I to IV), but only version I has been used in the clinics. In the rest of this article, we will describe the European RBE model as LEM.

The LEM and MKM models are different in their physical and mathematical assumptions and rely on different reference endpoints (human salivary gland cell lines survival for MKM, and idealized chordoma cell lines survival for LEM). For a given plan, the nominal values of RBE-weighted dose differ systematically between the 2 models. This difference depends on several parameters, such as dose per fraction, size shape and position of the target volumes, and the number and direction of beams. Comparing results obtained with the 2 models is not straightforward; however, a methodology has been established for that comparison and has allowed the use of Japanese protocols (validated by clinical data) in the European setting and with LEM model [12–14].

In the rest of the article, to avoid any possible confusion, we will always specify which RBE model we are referring to, explicitly stating it either as Gy RBE (LEM) or as Gy RBE (MKM).

## CIRT Protocols for Head and Neck in MedAustron

The CIRT has been used to treat several tumors of the head and neck area. It has not been employed for squamocellular carcinoma—the most common entity—but rather, for other, radioresistant histologies, such as salivary gland tumors [15–22], paranasal sinuses tumors (including sinonasal undifferentiated carcinoma, sinonasal adenocarcinoma, intestinal-type adenocarcinoma, and esthesioneuroblastoma, among others) [15, 23], mucosal melanoma [24–28], lacrimal gland tumors [29–31], and bone and soft tissue sarcoma (including tumors of the skull base and cervical spine [32–38]

At MedAustron, CIRT was begun in July 2019. Until September 2020, 91 patients have been treated and 42 of them had tumors in the head and neck.

The RBE model employed is that of the LEM version I with standard free parameters: α_γ_, 0.1 Gy^−1^; β_γ_, 0.05 Gy^−2^; *D_t_*, 30 Gy; and nuclear radius, 5 μm.

Dose and fractionation follow either the established experience of Japanese centers (with the conversion from MKM to LEM RBE [12–14] or the established German experience. The published results, number of treated patients, and length of follow up have been considered for this strategic decision. For mucosal melanoma and sarcomas (except skull base chordomas), we follow the Japanese fractionation schedule. For skull base chordoma, we follow the German approach. For salivary gland and paranasal sinus tumors, we follow the Japanese or German approach based on the need for elective nodal irradiation (ENI).

Although ENI is not routinely performed with CIRT, when ENI is indicated it is performed with photons in a regional radiotherapy department, and CIRT is employed as a boost. Table 1 summarizes the dose and fractionation employed in MedAustron.

**Table 1.** Summary of the dose and fractionation employed in MedAustron Ion Therapy Center (Wiener Neustadt, Austria).

**Disease**	**Total dose, Gy RBE (LEM)**	**Dose/fraction, Gy RBE (LEM)**	**Total No. of fractions**	**Fractions/wk**	**Comment, schedule**
Mucosal melanoma	65.6–68.8	4.1–4.3	16	4	Japanese
Salivary glands (no ENI)	65.6–68.8	4.1–4.3	16	4	Japanese
Paranasal sinuses (no ENI)	65.6–68.8	4.1–4.3	16	4	Japanese
Sarcoma, except skull base chordoma	76.8	4.8	16	4	Japanese
Skull base chordoma	66	3	22	5	German
Salivary gland with ENI (low-LET photon RT up to 50 Gy, then CIRT boost)	24	3	8	5	German
Paranasal sinuses with ENI (low-LET photon RT up to 50 Gy, then CIRT boost)	24	3	8	5	German

**Abbreviations:** Gy RBE, Gy relative biological effectiveness; LEM, local effect model; ENI, elective nodal irradiation; RT, radiotherapy; CIRT, carbon ion radiotherapy; LET, linear energy transfer.

## OAR Constraints at MedAustron

Historically, head and neck tumors have been treated with CIRT, if they had intracranial extension and/or involved the skull base macroscopically. Rarely have tumors of larynx or oro-hypopharynx undergone CIRT. This explains the paucity of data for those anatomic sites and explains why CIRT dose constraints for some organs (such as larynx and swallowing structures have not been investigated extensively. In this article, we will, therefore, focus on those OARs that are most relevant to CIRT in clinical practice and for which clinical data are available.

### Optic Nerve and Optic Chiasm

In the Japanese experience from the National Institute of Radiological Sciences (NIRS; Chiba, Japan), many patients were treated with a plan that exceeded the constraints for 1 optic nerve. That approach resulted in a significant number of clinical adverse events and made it possible to derive a dose-effect curve for optic neuropathy [39]. In 54 evaluable patients, no optic neuropathy was observed with a maximum dose (*D*_max_) < 57 Gy RBE (MKM). The logistic regression model identified a *D*_20%_ < 28 Gy RBE (MKM) and a *D*_1%_ < 40 Gy RBE (MKM) as totally safe dose constraints. In the initial experience at Centro Nazionale Adroterapia Oncologica (CNAO; Pavia, Italy), the Italian CIRT facility, these constraints were applied without attempting to convert them from MKM to LEM (which was a conservative approach because conversion to LEM would have made the constraints more permissive). Recently, the low incidence of optic neuropathy in the CNAO series and the recalculation of the LEM plan with MKM model has allowed those constraints to be relaxed to *D*_20%_ < 40 Gy RBE (LEM) and a *D*_1%_ < 50 Gy RBE (LEM) [40].

There is no published analysis of optic pathway toxicity from the German group. The reported constraint for optic pathways used at the former GSI Helmholtzzentrum für Schwerionenforschung, Darmstadt, Germany, and subsequently at the Heidelberg Ion Therapy (HIT; Heidelberg, Germany) is *D*_1%_ < 54 Gy RBE (LEM) [38].

Currently, at MedAustron sparing vision on both sides is a high priority. However, in certain clinical situations, we are forced to accept a clinically significant risk of unilateral vision loss. In those cases, we have selected a constraint for low-to-middle risk and a constraint for middle-to-high risk of optic neuropathy. When none of these constraints can be respected unilaterally, we consider the treatment as nonvision preserving for the affected side. A significant risk of bilateral vision loss is not acceptable. These constrains are derived from the Japanese and German experience (with the conversion from MKM to LEM performed by the Italian group); see Table 2.

**Table 2.** Dose constraints for optic pathways at MedAustron Ion Therapy Center (Wiener Neustadt, Austria).

**Fractionation**	**Safe constraint, Gy RBE (LEM)**	**Low-to-medium risk,^a^ Gy RBE (LEM)**	**Medium-to-high risk,^a^ Gy RBE (LEM)**
Japanese	*D*_1%_ < 50	*D*_1%_ < 54	*D*_1%_ < 57
	*D*_20%_ < 40	*D*_20%_ < 40	*D*_20%_ < 40
German	*D*_1%_ < 54	*D*_1%_ < 57	*D*_1%_ < 60

**Abbreviations:** Gy RBE, Gy relative biological effectiveness; LEM, local effect model, *D*, dose.

a Used for only one optic nerve, sparing chiasm and contralateral nerve.

### Brainstem

Brainstem toxicity is a much feared and potentially life-threatening toxicity. Understandably, every effort is made in clinical practice to prevent it and, as a result, few data points are available to analyze the dose-response curve.

The Japanese group from Gunma University specifically analyzed brainstem toxicity after CIRT [41].

The authors described asymptomatic contrast enhancement in the brainstem as grade 1 necrosis and reported a 3-year incidence of 6.5% for that endpoint. No grade 2 or greater toxicity was observed.

The constraints suggested by the Gunma group were V30 Gy RBE (MKM) of < 0.7 cm^3^ and a V40 Gy RBE (MKM) of < 0.1 cm^3^. These values are consistent with those used in other Japanese facilities. Those same values were initially used at CNAO with the LEM model, and no brainstem event was observed. A recalculation of LEM plans with the MKM model has been performed by CNAO colleagues [42] and has permitted a translation of the MKM values of 40 Gy RBE (MKM) and 30 Gy RBE (MKM) in the LEM values of 46 Gy RBE (LEM) and 38 Gy RBE (LEM), respectively.

There is no published analysis of observed brainstem toxicity from the German group. In clinical practice, the volume of brainstem receiving 50 Gy RBE (LEM) “has to be minimized,” and a maximum dose shall not exceed 54 Gy RBE (LEM) [8, 43].

Currently, at MedAustron, we use the constraints derived from the Japanese experience (with Italian conversion) and from the German experience (Table 3).

**Table 3.** Dose constraints for the brainstem at MedAustron Ion Therapy Center (Wiener Neustadt, Austria).

**Fractionation**	**Safe constraint, Gy RBE (LEM)**
Japanese	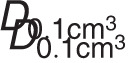 < 46
	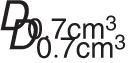 < 38
German	*D*_2%_ < 50
	*D*_max_ < 54

**Abbreviations:** Gy RBE, Gy relative biological effectiveness; LEM, local effect model, *D*, dose.

### Spinal Cord

Because of the lack of any specific analysis of spinal-cord toxicity after CIRT and following the clinical practice of proton facilities, such as Massachusetts General Hospital (Boston) and the Paul Scherrer Institut (Villigen, Switzerland), we apply the same constraints that we use for the brainstem to the cervical spine (Table 4).

**Table 4.** Dose constraints for spinal cord at MedAustron Ion Therapy Center (Wiener Neustadt, Austria).

**Fractionation**	**OAR constraint, Gy RBE (LEM)**
Japanese	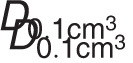 < 46
	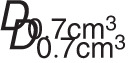 < 38
German	*D*_2%_ < 50
	*D*_max_ < 54

**Abbreviations:** Gy RBE, Gy relative biological effectiveness; LEM, local effect model, *D*, dose.

### Cochlea

To our knowledge, there are no available published data specifically on the subject of cochlea constraints and hearing loss after CIRT. Because of the physical properties of carbon ions (especially the small spot size and the sharp penumbra), it is relatively easy to spare a cochlea that is not included in the target volume.

At MedAustron, we use dose constraints that are derived from clinical practice and extrapolation from low-LET data (Table 5).

**Table 5.** Dose constraints for cochlea at MedAustron Ion Therapy Center (Wiener Neustadt, Austria).

**Fractionation**	**Safe constraint, Gy RBE (LEM)**	**Acceptable constraint, Gy RBE (LEM)**
Japanese and German	*D*_mean_ < 30	*D*_mean_ < 43

**Abbreviations:** Gy RBE, Gy relative biological effectiveness; LEM, local effect model, *D*, dose.

Those constraints are more conservative than the constraints reported by colleagues from HIT in the ICRU report [9], but in our experience, they are relatively easy to fulfill, at least on one side, without compromising target coverage.

### Brain Parenchyma

Brain necrosis is a serious toxicity that can potentially jeopardize quality of life and may lead to a patient's death. In several cases, after CIRT or proton therapy, patients develop radiological findings consistent with brain necrosis but do not report any symptoms. Most of those cases (designated as grade I according to Common Terminology Criteria for Adverse Events, version 5.0; US National Cancer Center, Bethesda, MD), resolve spontaneously. Analyzing brain necrosis focusing only on asymptomatic radiologic changes may lead to excessively conservative constraints; however, to estimate a dose-response relationship, imaging is mandatory, and the censoring of radiologically apparent, but clinically asymptomatic, case is a complex and potentially confounding issue but provides important data points.

The group from GSI/HIT in Germany has reported on 59 patients treated for skull base chordoma and chondrosarcoma with CIRT with a dose per fraction between 3 and 3.5 Gy RBE (LEM) [44]

A relatively high percentage of those patient (28% at 2 years) developed radiographic changes in the temporal lobe, but only one fifth of them reported any symptoms. The strongest dosimetric predictor of radiologic changes was the maximum dose to 1 cm^3^ after excluding the cubic centimeter receiving the highest dose (*D*_max_,V1 cm^3^). Doses were converted to the normalized total dose (NTD)_α/β=2Gy_, and the total dose resulting in 5% risk of toxicity (TD_5_) was 68.3 Gy RBE (LEM). In the Japanese experience, patients receiving high-dose CIRT for head and neck cancer had a high risk of developing radiologic findings consistent with necrosis. As reported by the NIRS group, those findings were self-limiting and did not require any treatment in > 40% of the cases [45]. The analysis performed at NIRS was, therefore, focused on symptomatic brain necrosis in patients treated with 16 fractions CIRT and a dose per fraction between 3 and 3.8 Gy RBE (MKM). The most significant parameter was the volume receiving > 50 Gy RBE (MKM), with 4.6 cm^3^ selected as optimal threshold [46].

A formal conversion of MKM to LEM constraints has not, to our knowledge, been performed; however, extrapolating from the prescription-dose conversion and from the brainstem cases, we estimated that 54 Gy RBE (LEM) could be considered a safe LEM equivalent of 50 Gy RBE (MKM). Thus, we think we have created a safe constraint as well as a low-to-medium risk constraint (Table 6) that can still reduce the risk of symptomatic necrosis but also acknowledge that, in many clinical conditions, even significant risk of localized brain necrosis is unavoidable).

**Table 6.** Dose constraints for brain parenchyma at MedAustron Ion Therapy Center (Wiener Neustadt, Austria).

**Fractionation**	**Safe constraint, Gy RBE (LEM)**	**Low-to-medium risk, Gy RBE (LEM)**
Japanese	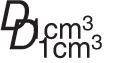 < 54	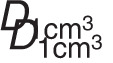 < 64
	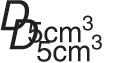 < 50	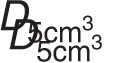 < 60
German	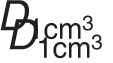 < 56.7 equivalent to NTD_α/β=2Gy_ = 65	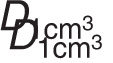 < 59 equivalent to NTD_α/β=2Gy_ = 69

**Abbreviations:** Gy RBE, Gy relative biological effectiveness; LEM, local effect model, *D*, dose; NTD, normalized total dose.

### Salivary Glands

Parotid glands are a relevant OAR for many classic CIRT indications, despite formal analysis of CIRT-induced xerostomia not yet being performed.

An article from NIRS [47] has analyzed the correlation between dosimetric parameters and parotid gland atrophy, as measured by comparing volumes before and after RT on imaging scans. According to this analysis the volume receiving > 5 Gy RBE (MKM) is the stronger predictor of atrophy. It is, however, clear that volume reduction is a suboptimal surrogate for parotid function and does not correlate perfectly with xerostomia.

At present, in MedAustron, we are simply applying photon-RT–derived dose constraints for this important organ (Table 7).

**Table 7.** Dose constraints for parotid glands at MedAustron Ion Therapy Center (Wiener Neustadt, Austria).

**Fractionation**	**Normal constraint, Gy RBE (LEM)**	**Constraint,^a^ Gy RBE (LEM)**
Japanese and German	*D*_mean_ < 26	*D*_mean_ < 20

**Abbreviations:** Gy RBE, Gy relative biological effectiveness; LEM, local effect model, *D*, dose.

a When the contra-lateral gland has to be sacrificed.

### Eye and Adnexa

Eye and adnexa are other important organs that can be considered “orphans,” because, until now, no specific CIRT toxicity analysis has been performed and no CIRT constraints have been set, to our knowledge. The only existing formal analysis focused on lacrimal duct dose constraints [48]; however, the lacrimal duct is rarely a clinical issue because it is typically either far away from, or well into, the target volume and would never be prioritized over target coverage.

At MedAustron, we have set constraints for lens, cornea, lacrimal gland, macula, and retina based on clinical judgments and extrapolation from photon experience. For each organ, 2 values are determined: a safe one and a more-permissive one corresponding to a low-to-medium risk of toxicity. Lens constraint can, if necessary, be exceeded because there is an excellent surgical salvage option for cataracts. Similarly, lacrimal gland constraints can be exceeded because even surgical resection of the gland does not necessarily results in severe xerophthalmia. If the constraint on the retina cannot be respected, we try to at least respect the constraint on the macula. These constraints, as shown in Table 8 are applied both for the German and for the Japanese fractionation.

**Table 8.** Dose constraints for eye and adnexa at MedAustron Ion Therapy Center (Wiener Neustadt, Austria).

**Organ**	**Safe constraint, Gy RBE (LEM)**	**Low-to-medium risk, Gy RBE (LEM)**
Cornea	*D*_2%_ < 30	D_2%_ < 40 and D_10%_ < 30
Lens	*D*_2%_ < 8	NA
Lacrimal gland	*D*_mean_ < 30	*D*_mean_ < 40
Retina	*D*_2%_ < 40	D_2%_ < 45
Macula	*D*_2%_ < 40	D_2%_ < 45

**Abbreviations:** Gy RBE, Gy relative biological effectiveness; LEM, local effect model, *D*, dose; NA, not available.

### Maxilla/Mandible

Osteoradionecrosis is a well-known, severe adverse side effect of high-dose RT. In the Japanese experience, maxillary osteonecrosis was reported in patients without direct tumor infiltration in the maxilla [49]. The presence of teeth and the dose were clear risk factors with a volume received of > 50 Gy RBE (MKM), being the most significant dosimetric parameter. The presence of teeth appeared to be of great relevance (hazard ratios, 11.3 versus 1.15 for V50). It is difficult to evaluate whether the presence of teeth is, by itself, a risk factor despite optimal oral hygiene or whether poor oral hygiene is a necessary cofactor. A formal analysis of mandible toxicity with CIRT has not, to our knowledge, been published.

In summary, the risk of inducing osteoradionecrosis is multifactorial and multiple previous surgeries; infiltration by the tumor and poor dental status all add to increase the risks.

At MedAustron, we decided to apply bone constraints only for the more-aggressive Japanese fractionation and to use the same constraints for mandible and maxillary bone. We extrapolated the MKM/LEM conversion from analogous cases and used more-conservative values for the teeth-bearing portion of the bone (Table 9).

**Table 9.** Dose constraints for mandible and maxilla at MedAustron Ion Therapy Center (Wiener Neustadt, Austria).

**Organ**	**Safe constraint, Gy RBE (LEM)**	**Low-to-medium risk, Gy RBE (LEM)**
Mandible ramus/maxilla (except processus alveolaris)	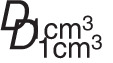 < 53	NA
	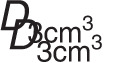 < 50	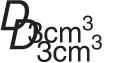 < 60
	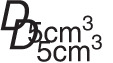 < 38	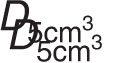 < 50
	NA	*D*_8cm3_ < 38
Mandible corpus/processus alveolaris	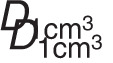 < 50	NA
	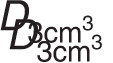 < 45	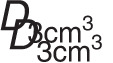 < 58
	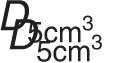 < 35	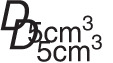 < 48
	NA	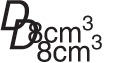 < 38

**Abbreviations:** Gy RBE, Gy relative biological effectiveness; LEM, local effect model, *D*, dose; NA, not available.

## Discussion

The specificity of CIRT demands a separate ad hoc investigation of dose constraints. This process is still ongoing. Because all CIRT facility have a research-oriented character, follow-up data of treated patients is readily available at each institution (including, in most cases, follow-up imaging). Conversion between the 2 clinically used RBE models is becoming available in commercial treatment planning systems, thus, significantly simplifying the joint analysis of Japanese and European results. Future developments should depend on 3 main activities: (1) extending and validating institutional dose constraints by formally analyzing observed clinical toxicity, (2) pooling multi-institutional data to increase statistical power (especially relevant for rarely observed toxicities) and further validating the RBE conversion method, and (3) interacting with the QUANTEC community to discuss the possibility of a carbon-ion subsection in the planned next QUANTEC revision.

To our knowledge, this article is the first attempt to formally report dose constraints used in a CIRT facility. We encourage similar publications by other institutions and to expand this process to include additional anatomic sites and organs.

Pooled data analysis is burdened with well-known technical, organizational, and legal issues. The research project HITRIplus (project number 101008548) [50] has been recently funded by the European commission within the framework of Horizon 2020. In this project, among many other activities, we foresee the setup of an infrastructure for pooled patient analysis among European CIRT centers, specifically focusing on OARs dose constraints.

Publication of institutional dose constraints for CIRT will be beneficial for upcoming facilities and will facilitate cooperation among existing centers. Pooled data analysis and review of routinely used constraints may significantly increase both the safety and the efficacy of CIRT.

## Conclusion

In this work, we describe CIRT dose constraints used in MedAustron in the head and neck area. Validation of dose constraints should present a relevant part of the activity of any CIRT facility, and we anticipate future multicentric joint evaluations.
